# High Prevalence of Vitamin D Deficiency in Newly Diagnosed Acute Myeloid Leukemia Patients and Its Adverse Outcome

**Published:** 2017-07-01

**Authors:** Fatere Seyedalipour, Ava Mansouri, Mohammad Vaezi, Kheirollah Gholami, Kazem Heidari, Molouk Hadjibabaie, Ardeshir Ghavamzadeh

**Affiliations:** 1 Clinical Pharmacy Department, School of Pharmacy, Tehran University of Medical Sciences, Tehran, Iran; 2 Research Center for Rational Use of Drugs, School of Pharmacy, Tehran University of Medical Sciences, Tehran, Iran; 3 Hematology-Oncology and Stem Cell Transplantation Research Center, Shariati Hospital, Tehran University of Medical Sciences, Tehran, Iran

**Keywords:** Acute myeloid leukemia (AML), Vitamin D, Remission, Relapse

## Abstract

**Background: **Although several studies have supported a preventive and therapeutic role of vitamin D (Vit D) for different types of cancers, we face insufficient documentation in acute myeloid leukemia (AML). So, we examined whether the serum calcidiol (25(OH)D) levels at the time of induction therapy have any impact on response and relapse in AML patients.

**Materials and Methods:** Blood samples were collected from 65 patients on days 0 and 28^th^ of treatment to evaluate serum concentration of 25(OH)D and its effects on complete remission (CR) achievement, relapse rate and hospitalization length.

**Results:** Of the 65 patients who were included in the study, 38 were male (58.5%) and 27 were female (41.5%). Median age at the time of treatment was 37 years (range 15-68). 6% of the participants were older than 60 years. In regard to 25(OH)D levels, 81.5% of AML patients were deficient (levels <20 ng/ml). There was a significant difference in CR between patients with sufficient and deficient level of 25(OH)D. Deficient patients had longer length of hospitalization than those with sufficient levels. Also Vitamin D deficient patients had higher serum ALP levels. The mean level of 25(OH)D on treatment day 28^th^ in our study was significantly lower than the baseline value.

**Conclusion:** The results of the study showed that serum 25(OH)D levels deficiency was highly prevalent among Iranian AML patients. Furthermore, higher Vit D levels in AML patients were associated with better outcome in these patients.

## Introduction

 Acute myeloid leukemia (AML) is caused by reduced capacity of myeloid precursors to differentiate into more mature cellular elements. Consequently, accumulation of leukemic blasts in the bone marrow causes reduction in the production of normal red blood cells, platelets, and mature granulocytes which leads to anemia, bleeding, and an increased risk of infection ^1^^-^^3^ . Eight major variants of AML are defined by the French-American-British (FAB) classification system based on morphologic characteristics (AML m0-7)^4^. AML-M3 also known as acute promyelocytic leukemia has different treatment from other AML subtypes^   3 ^. After AML (non- m3) diagnosis, induction chemotherapy is given to clear the bone marrow and peripheral blood of all blast cells in order to regenerate normal blood cell components ^5^^,^^6^ . Twenty to 30% of young adult patients and 50% of older adult patients with newly diagnosed AML will fail to attain a complete response (CR) with intensive induction chemotherapy as a result of drug resistance. In addition, a large percentage of patients who initially attain a CR will relapse. The therapy that provides the best chance for cure of patients with relapsed or refractory AML is allogeneic hematopoietic cell transplantation ^7^^-^^9^ .

Vitamin D (Vit D) is a fat-soluble vitamin that is produced by the human skin (the major source of VitD) in response to solar ultraviolet-Bradiation (UVB) and very little amount supplied from the food^   10 ^. In addition to significant clinical role in skeletal health, Vit D potentially regulates many other cellular functions. Extra skeletal effects of Vit D on cellular differentiation, controlling proliferation, apoptosis and immune modulation have been demonstrated^11^^,^^12^. Vit D is metabolized in the liver to 25-hydroxyVit D (25(OH)D),the major circulating metabolite- and further in the kidney, to 1,25-dihydroxyVit D (1,25(OH)_2_D), the bioactive form of Vit D. The measurement of 25(OH)D metabolite in serum is a clinical tool for determining Vit D status^   13 ^.

Vit D deficiency is very common around the world. In the National Health and Nutrition Examination Survey (NHANES 2005-2006), 41.6 percent of adult participants (≥20 years) had 25(OH)D levels below 20 ng/mL ^14^^,^^15^ .

Epidemiologic studies have suggested an association between Vit D deficiency and AML ^16^^-^^18^ . In addition, in the early 1980s, in vitro data showed the ability of 1,25(OH)_2_D to differentiate AML cells into mature myeloid cells^   19 ^. Several studies have shown that low serum 25(OH)D levels were associated with inferior event-free survival and overall survival in diffuse large B- and T-cell lymphoma patients^   20 ^ and decreased time until initiation of treatment in newly diagnosed chronic lymphoblastic leukemia (CLL) patients^   21 ^.

The following values were used to classify Vit D status; Vit D deficiency for values of 20 ng/mL or less; Vit D insufficiency for values between 20.1 and 29.9 ng/mL, and normal state (Vit D sufficiency) for values of 30 ng/mL or above ^11^^, ^^22^ .

Based on the above considerations, we prospectively tested the hypothesis that serum 25(OH)D levels prior to start of treatment have impact on response and relapse in patients diagnosed with AML. 

## MATERIALS AND METHODS

**Study design**

The present study was designed as a prospective cohort study to determine (for the first time in Iran) the levels of 25(OH)D in Iranian AML-diagnosed population. This cohort was evaluated duringthe 1-year period between February 2015 and March 2016 at the Hematology, Oncology and Stem Cell Transplantation Research Center, Shariati Hospital affiliated with Tehran University of Medical Sciences, Tehran, Iran. The study protocol was approved by the Research Ethics Committee of Tehran University of Medical Sciences (TUMS), and followed the principles outlined by the Helsinki Declaration. Informed consent was obtained from all individual participants included in the study.

**Patients**

All new diagnosed AML patients above 15 years old admitted to the Hematology-Oncology Unit, Shariati Hospital between February 2015 and March 2016 were included in the study. Patients with AML-M3, patients with resistant or relapsed AML, secondary AML caused by cytotoxic drugs or radiotherapy and history of hematologic disease such as myelodysplastic syndrome (MDS) or myeloproliferative disorder were excluded from the study. Finally, 65 patients were included in the study, and followed up 6 months after initiation of induction chemotherapy.

**Overview of treatment**

All AML patients received intensive first-line therapy with "7+3" regimens, which combined a 7-day continuous intravenous (IV) infusion of cytarabine (100 or 200 mg/m2/day) with an anthracycline (daunorubicin 90 mg/m²/day on days 1 to 3 as an IV bolus injection). The most commonly used anthracycline in this regimen was daunorubicin, but idarubicin might be used instead. The initial response to treatment was evaluated 7 to 10 days after the final doses of induction chemotherapy with a unilateral bone marrow aspirate and biopsy to demonstrate adequate marrow hypoplasia. A subsequent bone marrow exam was performed after recovery of neutrophils and platelets to document the remission status. Patients in complete remission (CR) received consolidation with "5+2" regimens (5 days cytarabine + 2 days daunorubicin at the same doses).

**Goal of treatment**

The traditional goal of treatment of AML was to produce and maintain a complete remission. The criteria for complete remission were platelet count higher than 100,000 cells/*µL, *neutrophil count higher than 1,000 cells/µL, and bone marrow specimen with less than 5% blasts^        23 ^, which were evaluated on the 28^th^ day of treatment. Then patients were followed for 6 months to assess relapse of disease.

**Serum 25(OH)D levels measurement**

Deficiencies in Vit D levels were neither assessed nor treated routinely.Two fasting venous blood samples (5 mL) were taken from each patient on the day before initiation of induction chemotherapy regimen and day 28 after treatment. Blood samples were centrifuged and serum extraction was done. Serum samples were then frozen immediately in -80°C and serum 25(OH)D concentrations were measured at the end of the study period. Serum 25(OH)D concentrations were measured by ELISA (immunodiagnostic System, Louvain-la-Neuve, Belgium). Samples were diluted with biotin-labeled 25(OH)D. The diluted samples were incubated in microtiter wells, which were coated with highly specific sheep 25(OH)D antibody for 2 hours at room temperature before aspiration and washing. Enzyme-labeled avidin was added and complexed selectively to biotin, then color was developed using a chromogenic substrate. The absorbance of the stopped reaction was read in a microtiter plate reader and color intensity was inversely proportional to the concentration of 25(OH)D. The patients were divided into two groups based on level of 25(OH)D (values < 20 ng/mL and values ≥ 20ng/ml).

**Data collection**

Baseline patients' demographic data, including sex, age, weight, height and type of AML were collected from the patients' medical records and databases. Laboratory parameters, including serum electrolytes (Na+, K+, Mg+2 and Ca+2), renal function tests (serum creatinine and urea), liver enzymes (Alanine aminotranspherase, Aspartate aminotranspherase and alkaline phosphatase) liver function indexes(serum albumin and Inter National Ratio (INR)) and molecular tests (WT1, FLT3) were also evaluated at the time of recruitment. Outcome parameters (i.e. Complete Blood Cell (CBC), leukemic blasts, hospitalization length and relapse) were collected. 

**Outcome data**

Two serum concentrations of calcidiol (25(OH)D) were recorded for each patient (related to blood samples of days 0 and 28^th^ of treatment) as the optimal indicator of Vit D status. The endpoints chosen for this study were: achieving a complete remission, relapse rate during 6 months, disease-free for six months (patients who attained complete remission with first induction therapy and did not relapse within 6 months) and the length of hospital stay within the period of 6 months. Hospitalization time was calculated as the number of days spent in the hospital during induction treatment and 6-month follow-up.

**Statistical analysis**

Statistical analyses were performed using the SPSS (Statistical Package for the Social Sciences) version 21 software (IBM Corp., Armonk, NY, USA). Continuous variables were presented as mean ± 0standard deviation (SD) or median (IQR). Categorical variables were reported as frequencies (percentages). Differences between two groups (Vit D deficient and sufficient) for continuous variables were tested using unpaired Student’s t-test or Mann-Whitney test according to the characteristics of the data distribution. Differences for categorical variables were analyzed with the chi-square and Fisher’s exact tests. Correlations were sought using the Pearson’s and Spearman’s coefficients for variables of normal and abnormal distributions, respectively. For all analyses, a two-sided p-value < 0.05 was considered to indicate statistical significance.

## Results

 Of the 65 patients who were included in the study, 38 were male (58.5%) and 27 were female (41.5%). Median age at the time of treatment start was 37 years (range 15-68) and 6% of patients were older than 60 years. In regard to 25(OH)D levels, 53 (81.5%) of AML patients had levels <20 ng/ml, while 9 (13.8%) and 3 (4.7%) of patients had levels between 20.1 and 29.9 ng/mL and ≥30 ng/ml , respectively. In other terms, 95.3% of all evaluated AML patients showed suboptimal Vit D levels (<30 ng/ml). The mean of 25(OH)D levels in patient groups with deficiency was 10.47 ng/ml.

Clinical and molecular characterization of the 65 AML patients enrolled in this study is summarized in Table 1 in terms of their 25(OH)D levels. The patients were divided into two groups based on level of 25(OH)D (values < 20 ng/mL and values ≥ 20ng/ml). No significant differences in age, gender or body mass index (BMI) were observed between patients with normal or low 25(OH)D levels. Moreover, molecular characteristics (FLT3 and WT1) were not different in two groups.

There was no significant difference in the baseline clinical characteristics of patients with and without Vit D deficiency, except for alkaline phosphatase (ALP). Vit D deficient patients had higher serum ALP levels (P = 0.04).

Two patients were excluded from study due to discontinuation of their treatment in the first week. Twenty-one patients (33.3%) had bone marrow specimen with more than 5% blasts on the day 28 of treatment and failed to achieve complete remission and 42 patients (66.7%) attained a complete remission (CR) with intensive induction chemotherapy. Of the 42 patients who had responded to initial treatment, 10 (23.8%) patients relapsed within 6 months. 

Serum 25(OH)D levels regarding the response rate, relapse, disease-free for 6 months and length of hospitalization have been shown in Table 2.

The mean level of 25(OH)D on the 28^th^ day of treatment in our study was 12.8±8.5ng/ml which was lower than baseline (14.1±10.6ng/ml, P= 0.03).

**Table 1:** Clinical characteristics of patients in regard to their serum 25(OH)D levels

**Patient characteristics**	**whole cohort N=65**	**Deficient (<20 ng/ml)** **N=53 (81.5%)**	**Sufficient (≥20ng/ml) N=12 ** **(18.5%)**	**P**
Mean age (years)	37.6±13.6	35.8±12.3	45.3±17.0	0.09
Median age (years, range)	37(15-68)	36(15-64)	47(17-68)	
Gender				
Male n (%)	38	32 (60.4)	6 (50)	0.51
Female n (%)	27	21 (39.6)	6 (50)	
BMI (kg/m2)	24.2	23.9	25.5	0.33
FLT3 present n (%)	16(24.6)	14 (26.4)	2 (16.7)	0.71
WT1 mutated present n (%)	58 (89.2)	47 (88.7)	11 (91.7)	0.99
Serum calcium level (mg/dL)	8.24±0.66	8.24±0.64	8.27±0.77	0.54
Serum phosphorus level (mg/dL)	3.68±0.80	3.65±0.70	3.78±1.17	0.72
Serum magnesium level (mg/dL)	1.88±0.23	1.89±0.211	1.78±0.26	0.20
Serum sodium level (mg/dL)	140.51±2.88	140.41±2.62	140.91±3.910	0.69
Serum potassium level (mg/dL)	3.98±0.39	4.03±0.36	3.77±0.50	0.17
Serum creatinine level (mg/dL)	0.86±0.19	0.85±0.19	0.89±0.23	0.58
Blood urea concentration (mg/dL)	13.61±4.50	13.25±4.35	15.33±4.99	0.11
Serum Albumin level (mg/dL)	3.69±0.46	3.68±0.48	3.75±0.37	0.47
INR	1.22±0.19	1.21±0.18	1.26±0.23	0.56
Serum ALT level (mg/dL)	29.12±17.24	29.34±18.00	28.16±14.05	0.96
Serum AST level (mg/dL)	24.83±11.17	25.47±11.43	22.00±9.90	0.42
Serum ALP level (mg/dL)	174.07±79.07	183.66±81.62	131.75±49.88	0.04*
Hemoglobin g/dL	8.57±1.79	8.57±1.79	8.53±1.89	0.71
Platelet count ×10^9^/L	63.13±84.48	51.89±44.07	112.83±170.41	0.44
WBC count ×10^9^/L	20.04±46.12	25.20±50.15	13.52±19.16	0.45

Lab data presented as mean ± Standard Deviation, 25(OH) D: 25 hydroxy vitamin D, ALT: Alanine aminotranspherase, AST: Aspartate aminotranspherase, BMI: Body mass index, ALP: Alkaline phosphatase, INR: International normalized ratio, WBC: White blood cell

* Significant difference

**Table 2:** Outcomes of patients in regard to their serum 25(OH)D levels

**Characteristics of Patients**	**whole cohort N=63**	**Deficient (<20 ng/ml)** **N=51 (81%)**	**Sufficient (≥20ng/ml) N=12 ** **(19%)**	**P**
Complete Remission n (%)	42(66.7)	31(60.8)	11(91.7)	0.04#
Relapse* n (%)	10 (23.8)	8(25)	2(18.1)	0.9
Disease-free for 6 months n (%)	32(50.7)	23(45.1)	9(75)	0.06
Median length of hospitalization(days, range)	40 (22-74)	44.5 (22-68)	32.5 (24-74)	0.04#

* Relapse in patients with initial CR (from 42 patients),

# significant difference

## Discussion

 This is the first study that reports the incidence of Vit D deficiency in Iranian newly diagnosed AML (non M3) patients and evaluates its effects on response to induction chemotherapy, the relapse of the disease in patients and disease-free for 6 months.

In this study, we found Vit D deficiency in most of the newly diagnosed AML (non M3) patients before the beginning of their intensive induction chemotherapy and on the 28^th^ day of treatment. We also found that the levels of 25(OH)D are significantly correlated with poor outcome.

Like other studies in healthy Iranian population and patients, we found that Vit D deficiency is more frequent in younger adults^24^^,^^25^. In the Middle East, predisposing factors of Vit D deficiency are inadequate sun exposure, lack of Vit D food fortification program, clothing habit, skin type, polymorphism in Vit D receptor, and lack of supplementation^   26 ^. But, since the use of Vit D supplements has received considerable attention in recent years among the elderly population, they mostly have sufficient levels of Vit D^   25 ^.

The median age at diagnosis of AML in previous studies was approximately 67 years^   27 ^, while the median age registered in our study was 37 years and several other studies also reported the lower age of AML diagnosis in Iran ^28^^, ^^29^ .

Looking at these two statements, we can draw the conclusion that there is a probable correlation between Vit D deficiency and AML occurrence in younger adults. 

Vit D deficiency has been reported to predispose individuals to increased risk of developing a number of cancers. Compelling epidemiological and experimental evidence support a role for Vit D in cancer prevention and treatment in many types of cancers^16^^-^^18^^, ^^30^ .

Several physiological and pathological conditions are related to an individual’s Vit D status. For example, chronic inflammatory conditions, liver and renal dysfunction and low serum albumin can affect 25(OH)D levels^   13 ^. These contributing factors have been ruled out by testing different lab parameters. 

In the present study, we have shown that baseline Vit D status before start of treatment is associated with the probability of CR attainment and CR rate is higher in patients with sufficient Vit D levels. In accordance with our study, several other studies demonstrated that low serum Vit D levels are associated with shorter survival in patients with myelodysplastic syndrome (MDS) and secondary oligoblastic AML^   31 ^ and worse outcome in (non-hodgkin's lymphoma) NHL(20)and CLL^   21 ^. Also,a cohort of 97 newly diagnosed AML patients treated on similar protocols (7+3+3) showed that patients with subnormal 25(OH)D3 (<32ng/ml) had significantly worse progression-free survival (PFS) and overall survival (OS) compared to those with normal 25(OH)D3 levels (≥32ng/ml)^32^. In vivo/in vitro studies as well as surveys of myeloid leukemia cell lines  have indicated that Vit D is generally capable of modulating several critical cellular processes, including inhibition of carcinogenesis by induction of cellular differentiation, inhibition of proliferation and promotion of apoptosis ^33^^-^^37^ . Vit D has other important effects such as the suppression of tumor angiogenesis, invasion and metastasis^   38 ^.

A significant inverse relationship was found between 25(OH)D levels and length of hospital stay. Similar findings were reported in other studies in critically ill patients and low serum levels of 25(OH)D was associated with increased time of ICU stay ^25^^,^^39^^,^^40^ . Likewise, in Radujkovic et al’s. study, lower VitD levels were associated with significantly longer hospitalization during treatment with azathioprin in patients with myelodysplastic syndrome and secondary oligoblastic AML due to more febrile neutropenia episodes^   31 ^. Low serum 25(OH)D leads to systemic inflammatory response syndrome, nosocomial bloodstream, respiratory tract infections, acute kidney disease, metabolic dysfunction and all-cause mortality ^41^^-^^43^ , which may cause prolonged hospitalization. In addition, in our study, lower rate of CR in Vit D deficient group and consequently the need for more intensive chemotherapy drugs could be associated with prolonged length of hospitalization.

Moreover, we have found that low levels of Vit D had significantly higher serum levels of ALP like other studies^   44 ^. Low 25(OH)D levels may be associated with secondary hyperparathyroidism and abnormal bone mineralization. Thus, increased levels of parathyroid hormone (PTH) and increased ALP levels should prompt suspicion of Vit D deficiency in some patients^   44 ^.

Another significant finding in this study was the difference between baseline levels of Vit D and day 28^th^ of treatment. In Fakih et al. study, chemotherapy was associated with a significant risk of severe Vit D deficiency in patients with colorectal cancer^   45 ^. It is plausible that patients undergoing chemotherapy less likely experience sunlight exposure, less able to absorb Vit D due to subclinical mucositis and less able to metabolize 25(OH)D into inactive compounds such as 24,25(OH)_2_Vit D through the activation of CYP3A4 or other metabolizing enzymes.

## CONCLUSION

 In conclusion, we found that deficiency in serum 25(OH)D levels was highly prevalent in Iranian AML patients. Furthermore, higher Vit D level in AML patients was associated with its better outcome. This study can provide a rationale for the design of clinical trials with larger sample size in order to evaluate the benefits of Vit D supplementation in deficient AML patients.

## References

[1] Döhner H, Estey EH, Amadori S (2010). Diagnosis and management of acute myeloid leukemia in adults: recommendations from an international expert panel, on behalf of the European LeukemiaNet. Blood.

[2] Meyers CA, Albitar M, Estey E (2005). Cognitive impairment, fatigue, and cytokine levels in patients with acute myelogenous leukemia or myelodysplastic syndrome. Cancer.

[3] Weber L, Stricker S, Williams C, Kirstein M (2013). Adult hematologic malignancies. Applied therapeutics: the clinical use of drugs.

[4] Bennett JM, Catovsky D, Daniel MT (1985). Proposed revised criteria for the classification of acute myeloid leukemia: a report of the French-American-British Cooperative Group. Ann Intern Med.

[5] Sekeres MA, Elson P, Kalaycio ME (2009). Time from diagnosis to treatment initiation predicts survival in younger, but not older, acute myeloid leukemia patients. Blood.

[6] Bertoli S, Bérard E, Huguet F (2013). Time from diagnosis to intensive chemotherapy initiation does not adversely impact the outcome of patients with acute myeloid leukemia. Blood.

[7] Wallen H, Gooley TA, Deeg HJ (2005). Ablative allogeneic hematopoietic cell transplantation in adults 60 years of age and older. J Clin Oncol.

[8] Gyurkocza B, Storb R, Storer BE (2010). Nonmyeloablative allogeneic hematopoietic cell transplantation in patients with acute myeloid leukemia. J Clin Oncol.

[9] Bertz H, Potthoff K, Finke J (2003). Allogeneic stem-cell transplantation from related and unrelated donors in older patients with myeloid leukemia. J Clin Oncol.

[10] Christakos S, Dhawan P, Verstuyf A (2016). Vitamin D: metabolism, molecular mechanism of action, and pleiotropic effects. Physiol Rev.

[11] Holick MF (2007). Vitamin D deficiency. N Engl J Med.

[12] Bikle D (2009). Nonclassic actions of vitamin D. J Clin Endocrinol Metab.

[13] Herrmann M, Farrell C-JL, Pusceddu I (2017). Assessment of vitamin D status–a changing landscape. Clin Chem Lab Med.

[14] Holick MF (2006). High prevalence of vitamin D inadequacy and implications for health. Mayo Clin Proc.

[15] Forrest KY, Stuhldreher WL (2011). Prevalence and correlates of vitamin D deficiency in US adults. Nutr Res.

[16] Timonen T (1999). A hypothesis concerning deficiency of sunlight, cold temperature, and influenza epidemics associated with the onset of acute lymphoblastic leukemia in northern Finland. Ann Hematol.

[17] Hassan IB, Islam SI, Alizadeh H (2009). Acute leukemia among the adult population of United Arab Emirates: an epidemiological study. Leuk Lymphoma.

[18] Boscoe FP, Schymura MJ (2006). Solar ultraviolet-B exposure and cancer incidence and mortality in the United States, 1993–2002. BMC cancer.

[19] Miyaura C, Abe E, Kuribayashi T (1981). 1α, 25-Dihydroxyvitamin D 3 induces differentiation of human myeloid leukemia cells. Biochem Biophys Res Commun.

[20] Drake MT, Maurer MJ, Link BK (2010). Vitamin D insufficiency and prognosis in non-Hodgkin's lymphoma. J Clin Oncol.

[21] Shanafelt TD, Drake MT, Maurer MJ (2011). Vitamin D insufficiency and prognosis in chronic lymphocytic leukemia. Blood.

[22] Holick MF, Chen TC (2008). Vitamin D deficiency: a worldwide problem with health consequences. The Am J Clin Nutr.

[23] Rowe JM, Neuberg D, Friedenberg W (2004). A phase 3 study of three induction regimens and of priming with GM-CSF in older adults with acute myeloid leukemia: a trial by the Eastern Cooperative Oncology Group. Blood.

[24] Heshmat R, Mohammad K, Majdzadeh S (2008). Vitamin D deficiency in Iran: A multi-center study among different urban areas. Iran J Public Health.

[25] Alizadeh N, Khalili H, Mohammadi M (2015). Serum Vitamin D levels at admission predict the length of intensive care unit stay but not in-hospital mortality of critically ill surgical patients. J Res Pharm Pract.

[26] Lips P (2007). Vitamin D status and nutrition in Europe and Asia. J Steroid Biochem Mol Biol.

[27] Siegel R, Naishadham D, Jemal A (2012). Cancer statistics. CA Cancer J Clin.

[28] Sanaat Z, Nouri M, Hajipour B (2011). Evaluation of copper, zinc, Cu/Zn, and VEGF in patients with AML in Iran. Iran J Cancer Prev.

[29] Ashrafi F, Shahnazari R, Samimi MA (2013). Results of treatment of acute myeloid leukemia in central part of Iran. Adv Biomed Res.

[30] Ma Y, Johnson CS, Trump DL (2016). Chapter Sixteen-Mechanistic Insights of Vitamin D Anticancer Effects. Vitam Horm.

[31] Radujkovic A, Schnitzler P, Ho AD (2017 Apr). Low serum vitamin D levels are associated with shorter survival after first-line azacitidine treatment in patients with myelodysplastic syndrome and secondary oligoblastic acute myeloid leukemia. Clin Nutr.

[32] Lee HJ, Muindi JR, Tan W (2014). Low 25 (OH) vitamin D3 levels are associated with adverse outcome in newly diagnosed, intensively treated adult acute myeloid leukemia. Cancer.

[33] Thompson T, Andreeff M, Studzinski GP (2010). 1, 25-dihydroxyvitamin D3 enhances the apoptotic activity of MDM2 antagonist nutlin-3a in acute myeloid leukemia cells expressing wild-type p53. Mol Cancer Ther.

[34] Jiang F, Bao J, Li P (2004). Induction of ovarian cancer cell apoptosis by 1, 25-dihydroxyvitamin D3 through the down-regulation of telomerase. J Biol Chem.

[35] Gocek E, Studzinski GP (2009). Vitamin D and differentiation in cancer. Crit Rev Clin Lab Sci.

[36] Nowak D, Stewart D, Koeffler HP (2009). Differentiation therapy of leukemia: 3 decades of development. Blood.

[37] Hughes PJ, Brown G (2006). 1α, 25‐dihydroxyvitamin D3‐mediated stimulation of steroid sulphatase activity in myeloid leukaemic cell lines requires VDRnuc‐mediated activation of the RAS/RAF/ERK‐MAP kinase signalling pathway. J Cell Biochem.

[38] Krishnan AV, Feldman D (2011). Mechanisms of the anti-cancer and anti-inflammatory actions of vitamin D. Annu Rev Pharmacol Toxicol.

[39] McKinney JD, Bailey BA, Garrett LH (2011). Relationship between vitamin D status and ICU outcomes in veterans. J Am Med Dir Assoc.

[40] Higgins DM, Wischmeyer PE, Sillau SH (2012). Relationship of vitamin D deficiency to clinical outcomes in critically ill patients. JPEN J Parenter Enteral Nutr.

[41] Kempker JA, Han JE, Tangpricha V (2012). Vitamin D and sepsis: An emerging relationship. Dermatoendocrinol.

[42] Ginde AA, Mansbach JM, Camargo CA (2009). Association between serum 25-hydroxyvitamin D level and upper respiratory tract infection in the Third National Health and Nutrition Examination Survey. Arch Intern Med.

[43] Ghashut RA, Talwar D, Kinsella J (2014). The effect of the systemic inflammatory response on plasma vitamin 25 (OH) D concentrations adjusted for albumin. PloS one.

[44] Kennel KA, Drake MT, Hurley DL, editors (2010). Vitamin D deficiency in adults: when to test and how to treat. Mayo Clin Proc.

[45] Fakih MG, Trump DL, Johnson CS (2009). Chemotherapy is linked to severe vitamin D deficiency in patients with colorectal cancer. Int J Colorectal Dis.

